# Novel Mechanisms of Spinal Cord Plasticity in a Mouse Model of Motoneuron Disease

**DOI:** 10.1155/2015/654637

**Published:** 2015-05-03

**Authors:** Rosario Gulino, Rosalba Parenti, Massimo Gulisano

**Affiliations:** ^1^Department of Biomedical and Biotechnological Sciences, University of Catania, Via Santa Sofia 64, 95127 Catania, Italy; ^2^IOM Ricerca s.r.l., Via Penninazzo 11, 95029 Viagrande, Italy

## Abstract

A hopeful spinal cord repairing strategy involves the activation of neural precursor cells. Unfortunately, their ability to generate neurons after injury appears limited. Another process promoting functional recovery is synaptic plasticity. We have previously studied some mechanisms of spinal plasticity involving BDNF, Shh, Notch-1, Numb, and Noggin, by using a mouse model of motoneuron depletion induced by cholera toxin-B saporin. TDP-43 is a nuclear RNA/DNA binding protein involved in amyotrophic lateral sclerosis. Interestingly, TDP-43 could be localized at the synapse and affect synaptic strength. Here, we would like to deepen the investigation of this model of spinal plasticity. After lesion, we observed a glial reaction and an activity-dependent modification of Shh, Noggin, and Numb proteins. By using multivariate regression models, we found that Shh and Noggin could affect motor performance and that these proteins could be associated with both TDP-43 and Numb. Our data suggest that TDP-43 is likely an important regulator of synaptic plasticity, probably in collaboration with other proteins involved in both neurogenesis and synaptic plasticity. Moreover, given the rapidly increasing knowledge about spinal cord plasticity, we believe that further efforts to achieve spinal cord repair by stimulating the intrinsic potential of spinal cord will produce interesting results.

## 1. Introduction

A feasible strategy for central nervous system (CNS) repair after injury or neurodegenerative diseases involves the activation of endogenous neural precursor cells (NPCs). Multipotent NPCs have also been isolated from the spinal cord (SC) [1–3]. These cells could be mobilized after SC injury (SCI), but their ability to generate neurons appears limited [2–5].

Another process promoting a functional recovery after SCI consists in plastic changes involving synaptic plasticity [6]. We have previously studied some mechanisms of SC plasticity, by using a mouse model of motoneuron depletion induced by intramuscular injection of the retrogradely transported, ribosome-inactivating toxin, cholera toxin-B saporin (CTB-SAP) [7–10]. In particular, we have demonstrated that synaptic plasticity could be responsible, at least in part, for the spontaneous recovery of locomotion after injury [10–15] and that brain-derived neurotrophic factor (BDNF) could exert a fundamental role in this process [12, 16–18].

Intrinsic and extrinsic molecular factors regulating adult neurogenesis have been widely explored [19–21]. Sonic hedgehog (Shh) is a secreted glycoprotein promoting proliferation of NPCs and their differentiation into neurons and oligodendrocytes, during both development and adulthood [22–25]. Notch-1 is a cell surface receptor working as a regulator of NPCs proliferation, cell fate, and dendritic and axonal morphology in embryonic as well as adult CNS [26–28], including SC [29, 30]. Numb is a signal transduction factor involved in stem cell maintenance and differentiation, as well as in neuritogenesis, by antagonizing Notch-1 signalling [31–34]. Noggin is a secreted glycoprotein involved in the embryonic morphogenesis. In particular, this protein induces neural tissue by acting as an inhibitor of bone morphogenetic proteins [35–37]. So far, little is known about the possible role of Noggin in the adult SC. It has been found that Noggin stimulates NPCs proliferation and synaptic plasticity in the hippocampus [38–40].

Interestingly, in our neurotoxic SCI model, Shh, Notch-1, and Numb proteins were also involved in events of synaptic plasticity linked to the spontaneous recovery of locomotion [10, 13]. Moreover, in the same model, Noggin appeared linked to the recovery of locomotion, but the mechanisms responsible for this effect remain unclear [13].

Transactive response DNA-binding protein of 43 kDa (TDP-43) is a nuclear DNA/RNA-binding protein involved in the regulation of transcription and RNA processing [41–43]. Recently, TDP-43 was identified into the cytoplasmic inclusions observed in amyotrophic lateral sclerosis (ALS), frontotemporal lobar degeneration, and Alzheimer disease [42, 44, 45], thus suggesting a putative toxic effect of TDP-43 aggregates. Recent findings suggest that some effects on motoneurons could be linked to the loss of function of the normal TDP-43 [42, 46–49]. Interestingly, it has been recently found that TDP-43 could be localized in the dendrites and may behave as a neuronal activity-responsive factor, probably affecting local RNA translation at the synapse [50–52]. Moreover, TDP-43 could have a role in motoneuron synaptic function [47, 53–56] and, in particular, it is probably involved in SC synaptic plasticity in our mouse CTB-SAP spinal lesion model [57].

In the present research, we would like to deepen the investigation of the role of the above-described factors in modulating the SC plasticity in a mouse model of neurotoxic motoneuron degeneration obtained by CTB-SAP lesion. For instance, the role of TDP-43 is particularly interesting, given its involvement in neurodegenerative diseases including ALS. Moreover, considering the described role of BDNF, Shh, Notch-1, Numb, and Noggin in the regulation of NPCs function and SC plasticity [10, 12, 13], we sought to characterize a functional model where these factors could collaborate in modulating the spontaneous SC plastic changes and the resulting functional recovery.

After histological and functional characterization of the model, compensatory changes within the SC, such as recovery of locomotion and cell proliferation, were evaluated in relation to the expression levels of TDP-43, BDNF, Shh, Notch-1, Numb, and Noggin.

## 2. Materials and Methods

Young adult male mice (*n* = 37) (Charles River, Strain 129, 5 weeks of age) were used. Animal care and handling were carried out in accordance with the EU Directive 2010/63/EU. All experiments have been approved by our institutions as well as by the Italian Ministry of Health. All efforts were made to minimize the number of animals used and their suffering. Surgical procedures were performed under deep anaesthesia where necessary (isoflurane).

### 2.1. Neurotoxic Lesion

Motoneuron depletion was induced by injection of CTB-SAP (Advanced Targeting Systems, San Diego, CA, USA) into the medial and lateral gastrocnemius muscles at a dose of 3.0 *μ*g/2.0 *μ*L PBS per muscle, as described previously [10]. After the bilateral injection of the toxin, mice were allowed to survive for either one week (LES-1 wk, *n* = 10) or one month (LES-1 mo, *n* = 8). Other animals received an equal volume of CTB-only vehicle, and they were then sacrificed at the same time points as lesioned ones (SHAM-1 wk, *n* = 3; SHAM-1 mo, *n* = 3). In order to perform histological and immunohistochemical evaluations of the effects of neurotoxin lesion, six mice were injected unilaterally and then transcardially perfused at either one week (*n* = 3) or one month (*n* = 3) after the lesion. The efficiency of CTB-SAP in producing selective motoneuron depletion after injection in the target muscle has been proven [7–10], thus providing an effective model of primary neurodegeneration. Similar methods of selective neurotoxic lesion have been already used in our laboratory [58, 59]. Finally, a group of animals were left untreated and used as normal controls for western blot experiments (NC; *n* = 7).

### 2.2. Functional Test

All bilaterally lesioned, as well as SHAM and NC, animals were subjected to grid walk test to evaluate the effects of lesion upon motor activity. Briefly, tests were performed blind to animal treatment, starting the day before lesion and then repeated at one week and at one month after toxin injection. Mice had to walk across a 50 cm long runway made of round metal bars placed at variable distance and moved at every trial to prevent habituation. The animals had to cross the runway three times per session. The number of footfalls relative to both hindlimbs at every crossing of the runway was counted and divided by the corresponding number of steps. Then, we calculated the average values between test repetitions.

### 2.3. Histology, Immunohistochemistry, and Microscopy

In order to evaluate the effects of the toxin on motoneuron depletion as well as on cell proliferation and glial reaction, the unilaterally CTB-SAP injected animals were perfused transcardially with phosphate-buffered 4% paraformaldehyde (pH 7.4). The lumbar SC was dissected out, postfixed for 1 hour, and then soaked overnight into a phosphate-buffered 20% sucrose solution at 4°C. Then, 20 *μ*m thick horizontal sections were cut on a freezing microtome and collected into three series subsequently used for immunofluorescence by using the following primary antibodies: mouse anti-ChAT (Immunological Sciences Cat. no. MAB10838; dilution 1 : 400) or mouse anti-glial-fibrillary-acidic-protein (GFAP) (Immunological Sciences, Rome, Italy; Cat. no. MAB16117; dilution 1 : 500). For double labelling, anti-Ki67 antibody (Abcam plc, Cambridge, UK; Cat. no. AB15580; dilution 1 : 200) was used together with anti-GFAP. In brief, sections were mounted on gelatin-coated slides, incubated for 1 hour in 5% normal donkey serum and 0.3% Triton X100 in PBS and then overnight at room temperature with the primary antibody solution containing 0.3% Triton X100 and 2% normal donkey serum. As negative control, the primary antibody has been omitted in some sections. After rinsing in PBS, sections were incubated for 1 h with the appropriate Alexa Fluor 488 or 568 donkey anti-mouse, anti-rabbit, or anti-goat secondary antibodies (Life Technologies, dilution 1 : 500), in PBS plus 2% normal donkey serum and 0.3% Triton X100. Then, sections were washed in PBS and counterstained for 5 minutes with DAPI (Life Technologies, dilution 1 : 20000) in PBS. Slides were coverslipped with PermaFluor (Thermo) and stored at 4°C until microscopy. The observation of immunostained sections was carried out by means of a laser confocal microscope (Leica Microsystems S.p.A., Milan, Italy).

### 2.4. Western Blotting

After the last test session, animals were sacrificed by decapitation. Lumbar SCs were dissected out and homogenized as previously described [10]. For western blot quantification, 20 *μ*g of protein was separated on a 4–20% polyacrylamide gel and transferred to a nitrocellulose membrane. Membranes were blocked for 1 hour with 5% BSA and incubated for 2 h with the following primary antibodies: mouse anti-ChAT (Immunological Sciences Cat. no. MAB10838; dilution 1 : 400), goat anti-BDNF (Santa Cruz Biotechnology Inc., Cat. no. sc-33905; dilution 1 : 200), goat anti-Shh (Santa Cruz Biotechnology Inc., Cat. no. sc-1194; dilution 1 : 300), rabbit anti-Notch-1 extracellular domain (Upstate Biotechnology, Millipore group; Cat. no. 07-218; dilution 1 : 700), goat anti-Numb (Santa Cruz Biotechnology Inc., Heidelberg, Germany; Cat. no. sc-15590; dilution 1 : 100), mouse anti-GFAP (Immunological Sciences, Rome, Italy; Cat. no. MAB16117; dilution 1 : 600), rabbit anti-Noggin (Millipore, Cat. no. AB5729; dilution 1 : 1000), and rabbit anti-TDP43 (Cell Signaling, Cat. no. 3449; dilution 1 : 1000). Then, membranes were washed and incubated for 1 h with the appropriate peroxidase-conjugate goat anti-rabbit (Thermo Scientific group; Cat. no. 1858415; dilution 1 : 6000), goat anti-mouse (Thermo Scientific group; Cat. no. 1858413; dilution 1 : 6000), or rabbit anti-goat (Millipore, Cat. no. AP106P; dilution 1 : 10000) secondary antibodies. Peroxidase activity was developed by enhanced chemiluminescent substrate (Pierce Biotechnology Inc., Thermo Scientific group; Cat. no. 34075) and visualized on a film (Kodak). Then, the protocol was repeated for quantification of actin, using a mouse anti-actin primary antibody (Millipore, Cat. no. MAB1501; dilution 1 : 700) followed by a goat anti-mouse secondary antibody (Pierce Biotechnology Inc., Cat. no. 1858413; dilution 1 : 5000). The films were digitally scanned and the relative 300 dpi grayscale images were used for optical density measurement by using Scion Image software. Density values were normalized to actin levels measured in the same membrane. All assays were performed in triplicate.

### 2.5. Statistical Analysis

Differences between lesioned and control groups in western blot and grid walk test data were evaluated by one-way ANOVA followed by Bonferroni's post hoc test.

In order to assess whether the motor performance could depend on the expression levels of the analysed proteins, we used the following multivariate regression model:

(1) MP=β0+β1ChAT+β2GFAP+β3BDNF+β4Shh+β5Notch-1+β6Numb+β7Noggin+β8TDP-43+ε,

where MP is the predicted value of motor performance, the terms in square brackets are the mean optical densities values, as measured by western blot, *β*_0_–*β*_8_ represent the regression coefficients, and *ε* is the residual error. From this general model, we eliminated the nonsignificant terms by using backward stepwise regression. This procedure starts with the complete model and removes iteratively the least significant predictors until only significant variables remain. We used a restrictive *α* value (*α* < 0.05) for which a given variable was allowed into the model and selected only final models that explained at least 20% of the dataset variance (*R* ^2^ > 0.20) with a *P* value of the regression ANOVA less than 0.01.

Where appropriate, we used the following multivariate regression model to find significant models explaining correlations between each protein and the others:

(2) PredictedP=β0+β1P1+β2P2+β3P3+β4P4+β5P5+β6P6+β7P7+ε,

where [P1]–[P6] are the average optical density values of the analysed proteins, as measured by western blot; “Predicted[P]” is the predicted mean value of optical density relative to a given protein; *β*_0_–*β*_7_ represent the regression coefficients; and *ε* is the residual error. ChAT and GFAP protein expression levels have also been included in the model to account for any effect of motoneuron depletion and glial reaction. All analyses were performed by means of Systat 12 (Systat Software Inc.).

## 3. Results

All animals survived surgery except an animal belonging to the LES-1 mo group. Starting at two or three days after injection, all CTB-SAP injected mice showed a significant weakness of the hindlimb, although they were still able to walk and perform functional tests. SHAM lesioned and normal groups did not differ from each other in terms of motor performance and western blot data. Therefore, these groups were pooled together in a single control group (CTRL).

### 3.1. Motoneuron Loss and Cell Proliferation in the Lesioned Lumbar SC

**Figure 1:**
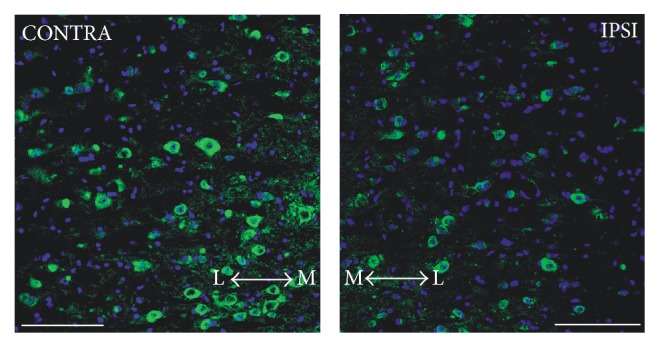
Fluorescence microscope images showing an example of lumbar SC section from a unilaterally lesioned animal, stained with anti-ChAT antibody plus 488 Alexa Fluor secondary antibody (green). Sections were counterstained with DAPI (blue) to visualize cell nuclei. The effect of neurotoxic lesion on the number of surviving motoneurons is evident in the right side of the cord, ipsilaterally to the injected muscle (IPSI), as compared to the contralateral side (CONTRA). Scale bar: 100 *μ*m. The arrows indicate the medial (M) to lateral (L) direction.

**Figure 2:**
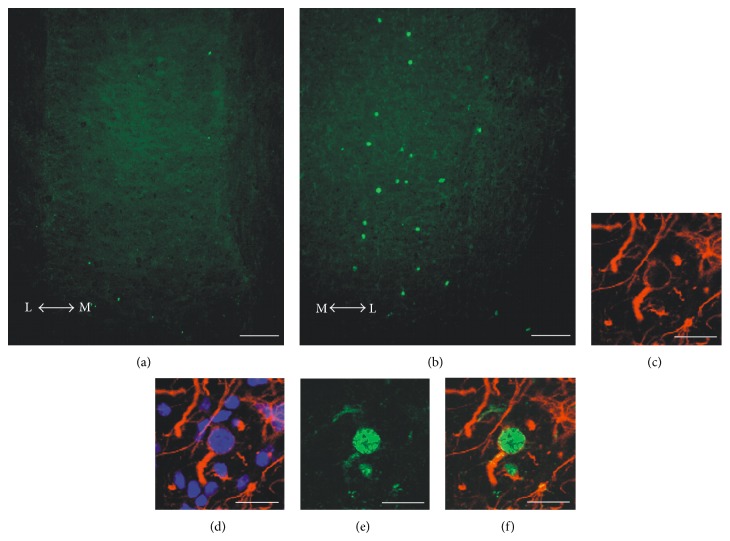
Panel of confocal images showing examples of immunostained lumbar SC sections collected from unilaterally lesioned mice. The panel shows the expression of Ki67 ((a), (b), green), in the right (lesioned) side (b), compared to the left (contralateral) side (a). The effect of CTB-SAP on cell proliferation is evident, although the number of proliferating cells is relatively low. Almost all the observed Ki67-positive cells ((b), (e), and (f), green) are also GFAP-positive ((c), (d), and (f), red). Sections were counterstained with DAPI to visualize cell nuclei (d). Scale bars: 100 *μ*m in (a), (b); 20 *μ*m in (c)–(f). The arrows indicate the medial (M) to lateral (L) direction.

The analysis of ChAT immunostained SC sections belonging to the unilaterally injected LES-1 wk group demonstrated evident motoneuron depletion within the lumbar SC ventral horn ipsilateral to the injected muscles, when compared to the contralateral side (Figure 1). This decrease (about 30%, as estimated by counting ChAT-positive motoneuron profiles in three horizontal sections per animal) was similar in animals sacrificed at one month after the lesion (not shown) and confirmed the results found in our previous studies [10, 12, 13]. Moreover, a population of Ki67-immunopositive profiles has been observed in the same area, ipsilaterally to the injected muscle (Figure 2(b)). Proliferating cells were conversely rare or absent in the contralateral side (Figure 2(a)). Confocal colocalization studies have shown that these cells are GFAP-positive astrocytes (Figures 2(c)–2(f)). Similar results have been found in animals killed one month after the lesion (not shown).

### 3.2. Modifications of Protein Expression after CTB-SAP Lesion

**Figure 3:**
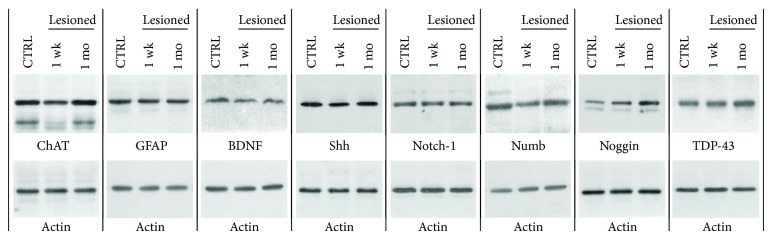
Western blots showing immunoreactive bands relative to ChAT, GFAP, BDNF, Shh, Notch-1, Numb, Noggin, and TDP-43, in relation to their corresponding actin signals in control and lesioned mice sacrificed at one week or one month after lesion.

**Figure 4:**
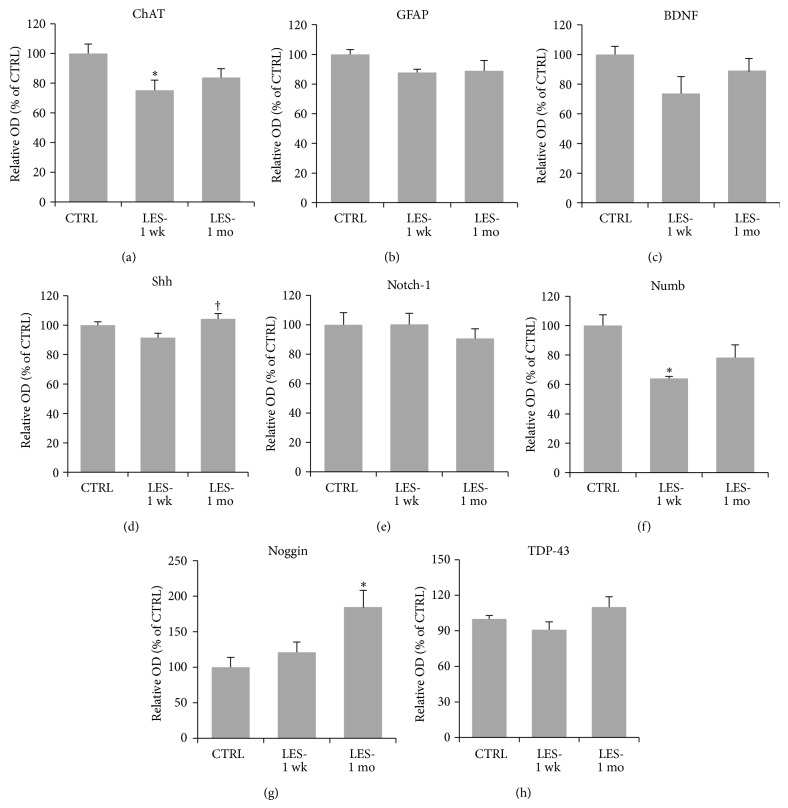
Graphs showing the average expression levels of proteins in SC homogenates from CTRL and lesioned animals analyzed at one week (LES-1 wk; *n* = 10) and one month (LES-1 mo; *n* = 7) after lesion, as measured by western blotting and normalized to actin levels. Values are mean ± s.e.m. They are expressed as percent of CTRL levels. Asterisks (∗) indicate significant difference from CTRL levels, whereas the dagger (†) indicates significant difference from LES-1 wk levels, as calculated by Bonferroni's post hoc test.

The analysis of western blot data revealed a significant (one-way ANOVA: *F*_2,27_ = 4.081, *P* = 0.028; Figures 3 and 4(a)) 25 ± 7% decrease of the average ChAT expression one week after the lesion (Bonferroni: *P* = 0.029; Figures 3 and 4(a)) and showed near-normal levels at one month (Bonferroni: *P* = 0.328; Figures 3 and 4(a)). The average expression levels of Shh one week after the lesion showed a small decrease (one-way ANOVA: *F*_2,27_ = 4.669, *P* = 0.018; Bonferroni: *P* = 0.104; Figures 3 and 4(d)) by 8 ± 3% compared to control levels, which was restored at one month (Bonferroni: *P* = 0.022; Figures 3 and 4(d)). Numb levels appeared significantly (one-way ANOVA: *F*_2,27_ = 8.511, *P* = 0.001; Figures 3 and 4(f)) reduced by 36 ± 1% at one week after lesion (Bonferroni: *P* = 0.001; Figures 3 and 4(f)), and they were partially but significantly restored at one month, being not significantly different from control levels (Bonferroni: *P* = 0.103; Figures 3 and 4(f)). The expression levels of Noggin were conversely significantly upregulated after lesion (one-way ANOVA: *F*_2,27_ = 5.155, *P* = 0.013). In particular, we found an increase of Noggin expression by 21 ± 1% at one week and by 85 ± 30% at one month, respectively. These levels were not significantly higher than control levels at one week (Bonferroni: *P* = 0.995; Figures 3 and 4(g)), but they became significantly higher at one month (Bonferroni: *P* = 0.011; Figures 3 and 4(g)). No statistically significant variations were found for GFAP (one-way ANOVA: *F*_2,27_ = 3.277, *P* = 0.053; Figures 3 and 4(b)), BDNF (one-way ANOVA: *F*_2,27_ = 3.067, *P* = 0.063; Figures 3 and 4(c)), Notch-1 (one-way ANOVA: *F*_2,27_ = 0.364, *P* = 0.698; Figures 3 and 4(e)), and TDP-43 (one-way ANOVA: *F*_2,27_ = 2.360, *P* = 0.114; Figures 3 and 4(h)).

### 3.3. Motor Deficits at the Grid Walk Test

**Figure 5:**
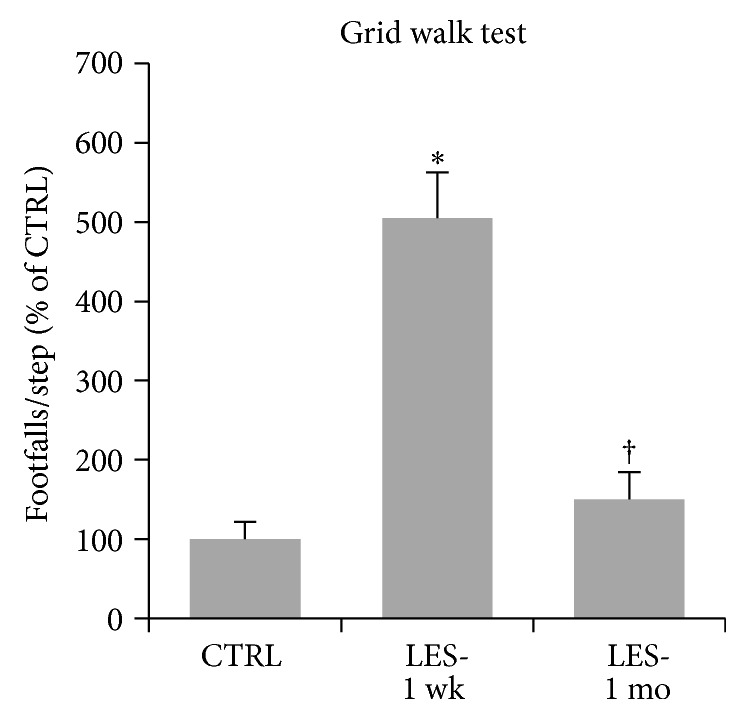
Motor performance scored at the grid walk test. Values are footfalls/step and are reported as mean ± s.e.m. They were normalized to control levels and expressed as percent of CTRL. Asterisk (∗) indicates significant difference from CTRL levels, whereas the dagger (†) indicates significant difference from LES-1 wk levels, as calculated by Bonferroni's post hoc test.

CTB-SAP lesion caused a significant effect on mice performance at the grid walk test (one-way ANOVA: *F*_2,27_ = 35.616; *P* = 0.000; Figure 5). One week after lesion, bilaterally lesioned mice showed a statistically significant fivefold increase of the number of footfalls/step compared with prelesion performance (Bonferroni: *P* = 0.000; Figure 5). Interestingly, one month after the lesion, animals showed a significant 3.4-fold recovery in the number of footfalls (Bonferroni: *P* = 0.000; Figure 5), reaching a grid walk performance significantly similar to prelesion levels (Bonferroni: *P* = 0.780; Figure 5).

**Figure 6:**
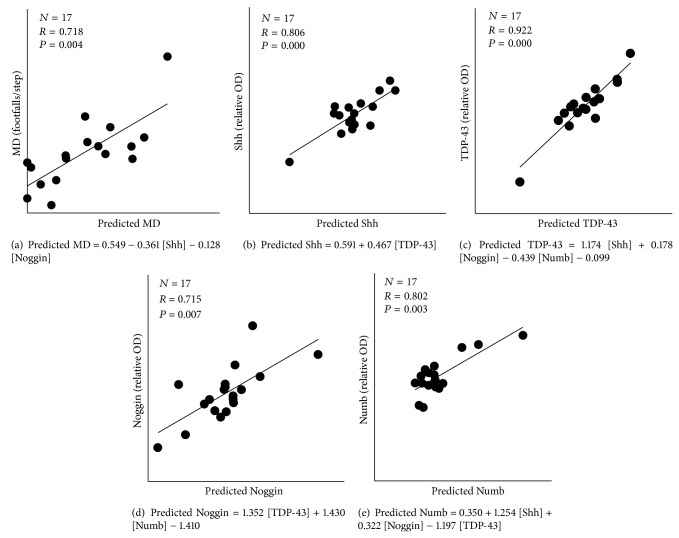
Significant correlations between the actual values of functional performance (a) or protein expression levels ((b)–(e)) with those predicted by the multivariate regression models after the application of backward stepwise regression. The final regression models represented in the graphs are reported in the grey window.

In order to address the possibility that the worsening of functional performance after injury and/or the following recovery could be linked to the protein expression levels, we used the multiple regression model in (1). LES-1 wk and LES-1 mo groups were pooled together and, after the application of backward stepwise regression, we obtained the model reported in Figure 6(a). The graph shows the highly significant correlation between the real values of grid walk performance and those predicted by the model (*R*_16_ = 0.718, *P* = 0.004; Figure 6(a)). It is evident that the expression levels of Noggin and Shh are good predictors of hindlimb performance and that they are inversely correlated with the number of footfalls. No association between variables was found within the control group (not shown).

Given the significant relationship between motor performance of lesioned animals and the expression levels of both Noggin and Shh, we again used a multivariate regression model (2) to verify if these proteins could be associated with each other. The results have shown that the expression of Shh could be predicted by TDP-43 levels (Figure 6(b)). The results have also shown that TDP-43 levels are directly linked to Shh and Noggin expression and inversely correlated with Numb expression levels (Figures 6(c) and 6(e)). Conversely, the expression levels of Noggin are directly linked to those of both TDP-43 and Numb (Figure 6(d)). No association between variables was found within the control group (not shown).

## 4. Discussion

In our previous studies [10, 12, 13], a neurotoxic SC lesion model was developed in order to study compensatory changes in the SC circuitry after selective motoneuron removal. The injection of CTB-SAP into the gastrocnemius muscle resulted in a partial depletion of lumbar motoneurons accompanied by the impairment of hindlimb function. The motoneuron loss was paralleled by a downregulation of ChAT within the lumbar SC at one week after the lesion. Given that the majority of acetylcholine release within the SC originates from motoneuronal activity, [60–62], it is likely that the observed downregulation of ChAT could be caused in large part by the motoneuron loss and in part by the consequent disruption of spinal circuitry. One month after the lesion, ChAT expression was partially restored, suggesting a partial recovery of the whole synaptic activity in the lumbar SC, which was accompanied by the restoration of motor performance, even though the motoneuron depletion is permanent. Therefore, an increased activity of the spared motoneurons could be responsible for both ChAT upregulation and functional recovery. On the other hand, the increased motoneuron activity could likely be supported by an upregulation of synaptic efficacy within the surrounding spinal circuitry. Similar to ChAT, Shh and Numb expression were downregulated at one week after the lesion and then restored at one month. Conversely, Noggin expression levels appeared gradually and significantly upregulated after the lesion. These results suggest that the expression of these proteins could be activity-dependent and probably linked to an increase of SC neuronal activity that compensate for the cell death and disruption of circuitry caused by the neurotoxin.

As demonstrated by the multivariate regression analysis, the observed changes of Shh and Noggin protein expression are inversely correlated with the number of footfalls at the grid walk test, thus suggesting that an artificial increase of their expression would improve motor performance in lesioned animals. Since Shh and Noggin affect functional performance of lesioned animals, we used multivariate regression analysis to verify if their expression is linked with those of the other proteins previously found to be involved in SC plasticity. The data have demonstrated that the expression levels of Shh are dependent on those of TDP-43. This finding is novel and interesting for a couple of reasons. In fact, it has been recently found that TDP-43 could regulate the local translation of mRNAs at the synapse, thus providing a complex modulation of synaptic strength [50–52]. Moreover, recent studies in our laboratory have demonstrated that TDP-43 could also modulate synaptic function by regulating the expression levels of both AMPA receptor subunits and synapsin-I [57]. Other recent findings have shown that the depletion of TDP-43 could cause synaptic effects and locomotor deficits in* Drosophila* [47, 53]. Interestingly, Shh seems to affect synaptic plasticity in a similar way [10, 12, 13, 63]. Therefore, the correlation between these proteins could be due to important functional association, which requires further investigation. Conversely, the mechanisms underlying the observed Noggin effect on motor activity are less clear. However, it is known that Noggin could stimulate functional recovery after SC injury by either enhancing axonal growth [64] or inducing neurogenesis [65]. Our results have shown that Noggin, whose expression levels increased after lesion, is strongly correlated with both TDP-43 (directly) and Numb expression (inversely). Moreover, Numb, whose expression levels were downregulated after lesion and then recovered in an activity-dependent manner, could also be modulated by either Shh (directly) or TDP-43 (inversely). The role of Numb in neurogenesis and synapse morphogenesis is well established [33, 34], whereas little is known about the possible role of Numb in mature neurons. However, it seems likely that this factor could participate in the axonal growth [31], as well as in the remodelling of dendritic spines and some aspects of synaptic function [66]. Here, we have shown that the Numb activity in the adult SC could take place in collaboration with other factors known to be involved in either neurogenesis and synaptic plasticity. The results collectively suggest differential roles of all these proteins in the SC, in either physiological or pathological conditions.

As demonstrated by Ki67 expression, a moderate cell proliferation occurs in the same SC area undergoing to motoneuron depletion. Colocalization studies have shown that almost all of these proliferating cells are GFAP-positive astrocytes. Glial reaction is a classical response to CNS injury that normally occurs as a result of tissue damage [67, 68]. Notably, our findings indicate that this process could also be caused by selective neurotoxic motoneuron degeneration, but its extent was limited, as demonstrated by the lack of GFAP increase after CTB-SAP lesion. This evidence is consistent with similar results obtained after neurotoxic lesion of cerebellar nuclei, which are also nonneurogenic CNS districts [69]. As previously introduced, Shh and Numb exert important roles in the regulation of adult neurogenesis [22, 24, 26–30, 33, 34]. Thus, it is reasonable to assume that the observed downregulation of Shh and Numb could be one of the factors involved in the glial reaction. Conversely, although the role of Noggin in stimulating neurogenesis in the adult brain is demonstrated [38, 39], its function in the lesioned SC is unclear. The observed Noggin increase after CTB-SAP injection could not likely be able to induce neurogenesis, but its effect in limiting glial reaction could not be excluded.

It is therefore likely that an experimental approach aimed at artificially modifying Shh, Numb, and Noggin signalling into the SC, during the first few weeks after the lesion, could enhance NPCs proliferation, reduce glial reaction, and remove some of the factors that inhibit neurogenesis. Moreover, the putative involvement of these factors in mechanisms of SC plasticity could anticipate a multiple positive effect of such treatments on the functional recovery.

Given the increasing interest in mouse models of TDP-43 gain or loss of function as models of neurodegenerative diseases, such as ALS animal models [70, 71], we believe that the elucidation of the physiological role of TDP-43 in the SC would provide an important contribution. Moreover, given the rapidly increasing knowledge about SC plasticity, we believe that further efforts to achieve SC repair by stimulating the intrinsic potential of SC will produce interesting results.
